# Highly Sensitive Temperature Sensing Performance of a Microfiber Fabry-Perot Interferometer with Sealed Micro-Spherical Reflector

**DOI:** 10.3390/mi10110773

**Published:** 2019-11-12

**Authors:** Jin Li, Juntong Yang, Jinna Ma

**Affiliations:** 1 State Key Laboratory of Nuclear Power Safety Monitoring Technology and Equipment, Shenzhen 518172, China; majinna@cgnpc.com.cn; 2 College of Information Science and Engineering, Northeastern University, Shenyang 110819, China; yangjuntongjiayou@163.com; 3 State Key Laboratory of Applied Optics, Changchun Institute of Optics, Fine Mechanics and Physics, Chinese Academy of Sciences, Changchun 130033, China

**Keywords:** microfiber, integrated optics, fiber sensors, temperature sensors

## Abstract

A temperature probe has been proposed by inserting a microfiber taper into a silica hollow core fiber with a microsphere end. The sealed air cavity in the microsphere and the inserted microfiber acted as the two reflectors of a Fabry-Perot interferometer, respectively. The contribution of both microfiber diameter and cavity length on the interference spectra was analyzed and discussed in detail. The temperature change was experimentally determined by monitoring the wavelength location of the special resonance dip. By filling the air cavity with poly-dimethylsiloxane (PDMS), a high temperature sensitivity of 3.90 nm/°C was experimentally demonstrated. This temperature probe with the diameter of 150 μm and length of 10 mm will be a promising candidate for exploring the miniature or implantable sensors.

## 1. Introduction

Temperature monitoring plays a very important role during chemical reaction, life health, industrial production, and environmental protection [1,2,3]. Fiber temperature sensors have always been a research hotspot, due to their many advantages compared with the traditional electrical temperature sensors, such as their light weight, small size, intrinsically safe, long-distance transmission, electromagnetic interference immunity, chemical corrosion resistance, high temperature resistance, etc. [4,5]. These unique properties pave their way to the possible applications in harsh environments and small space [6]. Fiber Fabry-Perot interferometers have been widely studied and used for temperature monitoring attributing to their smart structures and high resolution [7,8,9,10]. External temperature information can be obtained by demodulating the Fabry-Perot interference fringes formed by two optical signals with the phase difference depending on temperature change. Some works reported the Fabry-Perot interferometer temperature sensor and determined the temperature by the light intensity with the sensitivity of 60.79 nW/°C and a resolution of 0.5 °C, where the intensity was easily affected by the output stability of the light source [7]. The temperature sensitivity has been improved by using novel demodulation techniques, such as cursor effect, where two interference structures with different sensitivity or mechanics will be used to demodulate a sensitivity difference coefficient. Although the high sensitivity of 19.55 nm/°C was experimentally demonstrated by using liquid crystals and the Vernier effect, the demodulation process is complex, in which the locations of two resonance peaks/dips need to be traced and distinguished at the same time [8]. Fiber Fabry-Perot temperature sensors were implemented by mining different cavity or fusing different fibers. Two cascaded Fabry-Perot interferometers were fabricated by femtosecond laser, the sensitivities were experimentally demonstrated with 9.91 pm/°C during 100 °C to 400 °C and 15.88 pm/°C during 400 °C to 1100 °C [9]. Different kinds of fibers were cascaded fused together to form the Fabry-Perot interferometer, whose reflectors were played by the flat end-faces of fibers. A temperature sensor was reported by cascading a 200 μm thick silicon Fabry-Perot interferometer with another 10 μm thick silicon Fabry-Perot interferometer, where the sensitivity of 84 pm/°C was obtained [10]. By spliced one cut of fiber Bragg gratting (FBG) on one end of single mode fiber, a temperature sensitivity of 81.0 pm/°C was measured with the assumed resolution of 0.001 °C [11]. The Fabry-Perot cavities were fabricated at the fibers end by digging a hole or connecting a capillary and using the polymer film as the reflectors [12]. In some works, one of the reflectors was carried out using a flexible film coating at the end of open cavity, where the expansion of the film was easily affected by other environment parameters, such as humidity. For example, a Nafion film was prepared in the capillary to construct a Fabry-Perot structure to measure the temperature with the sensitivity of 2.71 nm/°C [13]. However, the environment humidity also exerted an impact on the wavelength shift. Mutilayers of the graphene diaphragm were covered as the reflector of one Fabry-Perot interferometer and experimentally demonstrated with a temperature-induced cavity length change of 352 pm/°C [14], but it will be difficult to precisely measure this length to explore the temperature values. Without introducing the sensitive materials, the fiber Fabry-Perot temperature sensors can operate at a temperatures up to 1200 °C with the sensitivity of 15.61 pm/°C [15]. However, the thermo-optic coefficient of the fiber materials was limited and resulted in a low sensitivity (13.6 pm/°C) [16]. The temperature sensitive materials will be used to fill the air cavity packaging temperature sensitive materials in the Fabry-Perot cavity [17]. These sensitive materials cover gas, liquid and cured solid ones, where the refractive index of gas or liquid will change significantly with the temperature change and cause the change in the light transmission distance, corresponding to the effective cavity length of Fabry-Perot interferometer [18], which is beneficial to improve the sensitivity and resolution of the fiber temperature sensor. The liquid polymer and the refractive index liquid contributed the sensitivities of 877 pm/°C [19] and 14.72 nm/°C [20], respectively. However, for the later one, the liquid was filled in a photonic crystal fiber. The liquid sensitive materials allow fiber sensors to have better temperature-sensitive characteristics, but also increase the cost and manufacturing difficulty. Poly-dimethylsiloxane (PDMS) has a high transparent and low refractive index, which results in its little impact on the incident light. Its high thermal expansion coefficient will enable a high sensitivity for monitoring the temperature change. Furthermore, it can be used as the filling materials in the liquid form and become solid later. The high sensitivity of up to 11.86 nm/°C was earlier reported in another work by using PDMS to fill the air gap [21].

This paper proposes a low-cost, simple and fast method for preparing fiber Fabry-Perot interferometer with miniature structures, which is composited by a microfiber taper, a hollow microtube sphere and PDMS. The Fabry-Perot interferometer was constructed by using the endface of a microfiber taper and the cone cavity surface of hollow microsphere fiber as its two reflectors. These two kinds of fiber structures can be easily fabricated using a burner or fiber splicer by flame melt drawing method and arc melting method, respectively. The temperature probe was fabricated by inserting the microfiber taper into the microtube hollow sphere and filling the PDMS solution into the air cavity. The length of the Fabry-Perot cavity was adjusted flexible by moving the microfiber taper in the microtube after filling the liquid formed PDMS. The perfusion and solidification of PDMS in the Fabry-Perot air cavity contributed to a high temperature sensing performance. A high temperature sensitivity of 3.9 nm/°C was experimentally demonstrated in this paper. Furthermore, the closed chamber of the hollow sphere can effectively eliminate the influence of gas and humidity in the environment to be measured.

## 2. Materials and Methods

### 2.1. Temperature Probe Design and Fabrication

**Figure 1:**
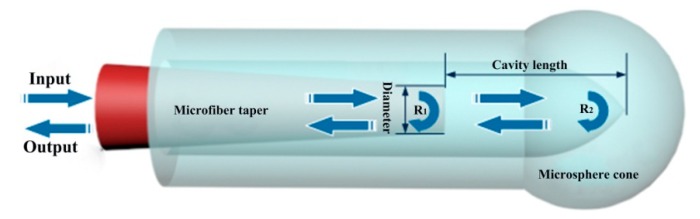
Schematic of microfiber-microsphere Fabry-Perot interferometer with a tunable cavity length by moving microfiber in a hollow core fiber based on micro-manipulation technique.

The designed Fabry-Perot interferometer structure is mainly composed of a microfiber taper and a hollow-core fiber, as shown in Figure 1. The two reflectors of the Fabry-Perot interferometer are acted by the end face of the microfiber taper and the cone surface of the hollow microsphere. When light is launched into the microfiber, a part of light signal will be reflected on the end face of the tapered microfiber, and another part of the reflection occurs on the cone surface of the spherical structure. These two-parts of reflected light beams have a different transmission distance (twice the cavity length) and interferes to each other forming an interference spectrum. The intensity and phase of the reflected light from the microfiber taper will be analyzed later by a spectrometer to explore their relationship with the temperature change.

Figure 1 indicates that the main parameters of this temperature sensor are the diameter of the tapered microfiber and the length of the cavity. The parameters selection will greatly affect the sensing performance. Therefore, it is necessary to compare and optimize the geometry parameters through experiments. The fiber Fabry-Perot structure will be packaged with the temperature sensitive material (PDMS), where the PDMS will be poured into the hollow fiber to fill the air cavity between the two reflective surfaces of the Fabry-Perot interferometer. Meanwhile, the microfiber taper will be fixed in the hollow core fiber. During the calibration of the temperature sensor, the packaged sensor needs to be placed in different temperature environments. When the temperature rises or changes, the cured PDMS is thermally expanded due to the thermo-optic effect and the elastic-optic effect, resulting in the cavity length change of the Fabry-Perot interferometer, as well as the reflecting spectra. By using this temperature sensor, the ambient temperature can be determined by tracking the phase change of the special resonance dips. 

**Figure 2:**
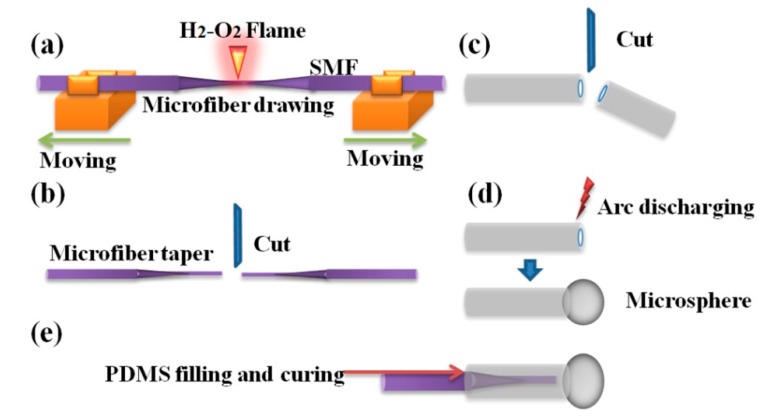
Fabrication process of a microfiber-microsphere Fabry-Perot interferometer temperature probe: (**a**) fiber drawing; (**b**) Microfiber taper preparing; (**c**) Silica hollow core fiber pre-treatment; (**d**) Microsphere fabrication; (**e**) PDMS filling and curing.

The complete fabrication process for the proposed Fabry-Perot interferometer is shown in Figure 2, including the fabrication of microfiber taper (Figure 2a), the diameters choice of tapered fiber (Figure 2b), the hollow core fiber pretreatment (Figure 2c), the preparation of the hollow spherical cavity (Figure 2d) and the PDMS filling and curing (Figure 2e).

To fabricate the microfiber taper, a normal single mode fiber (SMF, SMF-28, Corning Inc., Coring NY, USA) after removing the coating layer was fixed by two claps, point-heated by oxyhydrogen flame and stretched by a fiber melting-drawing system (IPCS-5000-ST, Idealphotonics Inc., Hong Kong, China). The diameter and length of the microfiber taper can be controlled by adjusting the flame temperature, gas volume and ratio, heating time and stretching speed. The microfiber taper was placed on the operation plate of a homemade fiber micromanipulation system to measure its diameter and precisely cut it at the desired region using a cutting pen under a microscope. The end face and diameter of the tapered microfiber were measured and recorded later. The hollow core fiber was chosen to prepare the hollow spherical structure by high-temperature melting method. Since the melting process needs to be completed in the fusion splicer, the size of the hollow fiber is limited. In our experiment, the outer diameter of the hollow core fiber is 150 μm (after removing the coating layer), and the inner diameter is only 100 μm (TSP100150, Polymicro Technologies, Inc., Phoenix AZ Arizona, USA). The coating layer (polyimide, melting point 350 °C) was firstly removed by the flame heating using a Bunsen burner, and then cleaned and placed in a fusion splicer. By using the manual welding operation model, the end of the hollow core fiber was located in the discharge zone, where the hollow spherical structure formed after arc discharge. The hollow core fiber was then cut at its other end with a suitable length to facilitate insertion of the microfiber taper. Finally, the temperature sensitive material PDMS was poured into the hollow core fiber to fill the Fabry-Perot cavity. The microfiber Fabry-Perot temperature probe was obtained after the PDMS becoming solidified. 

The microfiber Fabry-Perot interferometer structure has two main parameters, namely the microfiber taper diameter and the cavity length. The experimental results reveals that a thinner diameter (<20 μm) of the microfiber taper produces the multiple optical modes, which will increase the difficulty of demodulation process. The temperature will be determined by recording the wavelength shift of the resonance dip (finding the lowest intensity point in a free-spectra-range (FSR)), where the over-lapped dips will be difficult to distinguish. In this experiment, this diameter was controlled during 30–60 μm to obtain the ideal Fabry-Perot interference spectrum. The cavity length refers to the distance from the end face of the tapered fiber to the cone surface of hollow sphere. Here, it was precisely manipulated by moving the microfiber taper continually using a three-dimension fiber adjustment (APFP-XYZ, adjusting precision <2 μm, Zolix Instruments Co., Ltd., Beijing, China). The reflected spectra as a function of the cavity length were studied when the microfiber was fixed as a constant.

### 2.2. Cavity Length Optimization

**Figure 3:**
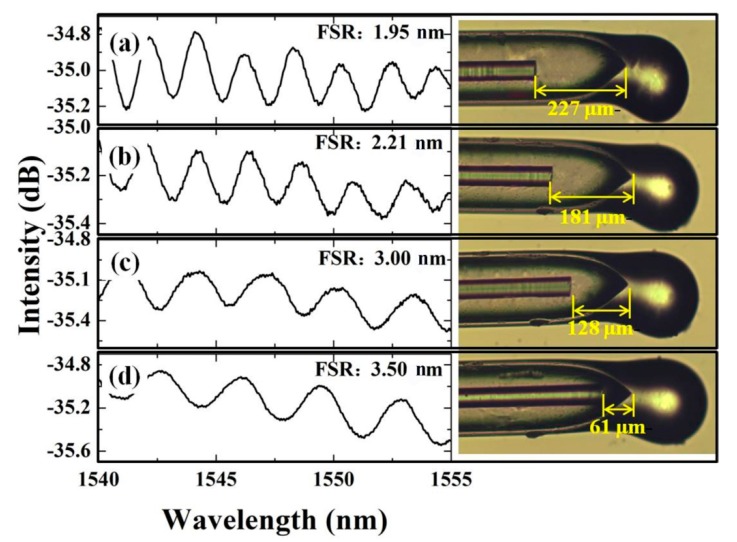
Reflected spectra of Fabry-Perot micro-interferometer with different cavity length (61 μm; 128 μm; 181 μm; 227 μm) by fixing microfiber taper in silica hollow core fiber.

The cavity length of the proposed Fabry-Perot temperature probe is easy to control before packaging. One can optimize it to meet the actual application needs (such as working range, sensitivity, sensor size) and obtain the desired optical properties, according to the quality of the reflection spectrum and the timely structure through CCD system equipped on a microscope (DMM-300C, Shanghai Caikon Optical Instrument Co., Ltd., Shanghai, China). The simple preparation process greatly saves costs and time. The appropriate cavity length range was selected based on analyzing the FSR and their extinction ratios (i.e., the ratio of the peak maximum power to the lowest power in the spectrum). To explore the impact of the cavity length of the F-P interferometer, the same microfiber with the diameter of 36 μm was used, and its position was adjusted in the hollow fiber to obtain the different cavity length. Figure 3 shows the reflectance spectra of a microfiber Fabry-Perot interference structure with the different cavity lengths of 61 μm, 128 μm, 181 μm, and 227 μm, respectively. Here, the cavity lengths were measured by the 2D micro-image measurement and analysis software (DMM-300, Shanghai Caikon Optical Instrument Co., Ltd., Shanghai, China). The light source is an amplified spontaneous emission (ASE, ASE-C light source, 1520–1560 nm, Shenzhen Golight Technology Co., Ltd.).

The spectra indicate that for a longer cavity length, the FSR becomes smaller, decreasing from 3.5 nm to 1.95 nm. Too small FSR will limit the operating range of the sensor. The temperature change information must be demodulated from resolving the wavelength position of a particular peak (dip). Therefore, a wavelength (phase) demodulation based sensor usually has one characteristic peak (dip) in its working range. In order to measure the temperature, one must find the peak (dip) position in the desired working range, also named FSR. During the wavelength (phase) demodulation process, the peak (dip) position in a FSR was determined by searching the highest (lowest) intensity point. The experimental results of Figure 3 indicate that a longer cavity will result in a short FSR and a sharp peak (dip) in the transmission spectra, which will further contribute a limited working range and a higher precision of a sensor. Conversely, a shorter cavity based sensor will have a wider working range, but the position of the peak (dip) will be more difficult to distinguish. Therefore, the cavity length should be balanced for designing a temperature probe. In this experiment, a temperature sensing probe with high sensitivity and precision is desired to prepare, whose cavity length will be minimized within the controllable range. The cavity length is controlled by ~40 μm, which makes it easier to fill in the PDMS solution.

### 2.3. Temperature Probe Package

**Figure 4:**
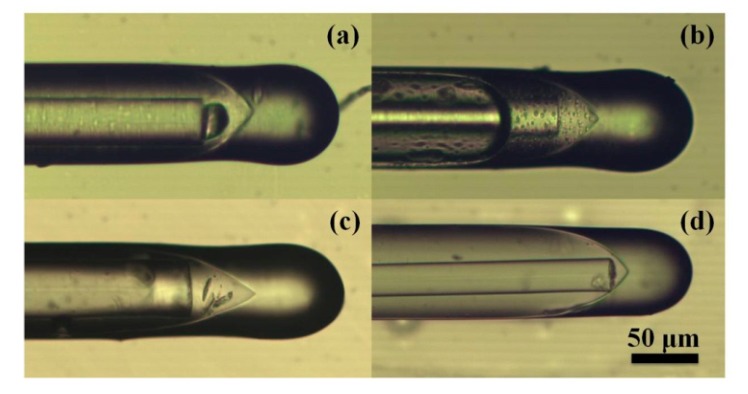
PDMS filled microfiber-air-cone with different defects (**a**–**c**) and non-defects (**d**). (**a**) big bubble; (**b**) small bubbles; (**c**) impurities.

After fixing the geometry parameters of the Fabry-Perot interference structure, the temperature sensitive material PDMS was filled to explore the temperature probe. During the initial stage of our experiment, the PDMS solution was configured using the ratio of the main agent: hardener: catalyst = 10:1:0.3. It is impossible to complete the filling process since the PDMS solution became solidified in a short time after the catalyst was added. Then, the PDMS solution with a ratio of primary agent: hardener = 10:1 was selected and poured into the microfiber Fabry-Perot structure. The catalyst was separately added later into the hollow core fiber and be heated at 80 °C for 1 h to cure it. Unfortunately, different defects or air bubbles were observed after curing the PDMS, as shown in Figure 4a–c, because it is difficult to evenly disperse the catalyst in the PDMS solution, resulting in the poor consistency of sensor fabrication, low sensitivity for temperature sensing (only 0.7569 nm/°C), and poor stability of structure.

Potting gel PDMS (SYLGARD 184, Dow Corning Co., Ltd., Michigan, USA) was finally used with a weight ratio of main agent: curing agent = 10:1. Unlike the original PDMS solution, it can be cured at 24 °C for 24 h or 80 °C for 1 h. Therefore, the time is sufficient to encapsulate the sensing structure at room temperature. This gel PDMS is a transparent fluid before curing and becomes a tough transparent elastomer after solidification with the excellent stability over a wide temperature range (−50 °C to 200 °C). The thermal conductivity is 0.17 ± 0.01 W/(m·K), and the elastic coefficient is 960 × 10^−6^. The good physical properties make it suitable as a temperature sensitive material for encapsulating the sensing structure proposed herein. After the PDMS solution was poured into the hollow core fiber, the position of the microfiber taper was adjusted in both radial and axial direction. By the assistance of the reflected spectra observing and geometry structure monitoring, the microfiber taper was finally fixed along the central axis of the hollow core fiber with the desired cavity length. The filled Fabry-Perot structure was placed in an incubator for 1 h at 80 °C to cure PDMS and obtain the temperature probe. As seen from Figure 4d, no small bubbles or other defects were generated in the filled cavity region. The microfiber Fabry-Perot temperature probe was finally obtained, containing a microfiber taper with the diameter of 50 μm, a cavity length of 41 μm, and a PDMS filling package length of 976 μm. The center axis of the microfiber taper coincided with that of the hollow core fiber. The interference spectrum was demonstrated with a good extinction ratio and FSR.

## 3. Results

**Figure 5:**
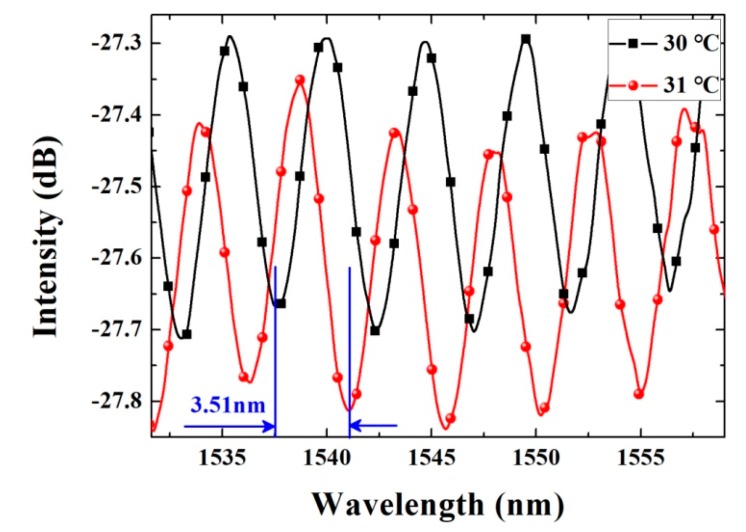
Resonance dip in the reflected spectra of microfiber Fabry-Perot cavity red-shifted for 3.51 nm when temperature increased from 30 °C to 31 °C.

To explore the temperature sensing performance, the microfiber Fabry-Perot temperature probe was placed in an incubator. When the temperature rises or falls, the cured PDMS will expand and its refractive index will change due to the thermo-optic effect and the elastic-optic effect, causing the change in both the Fabry-Perot cavity length and the cavity refractive index. Then the interference spectra will undergo red-shift or blue-shift. By tracing the shift direction and value of the special resonance peak/dip and demodulate its phase information, the according temperature change will be obtained. Figure 5 reveals the spectrum shift when the temperature changes from 30 °C to 31 °C. A resonance dip was chosen and continually traced to study its relationship depending on temperature change. 

When the temperature increased from 30 °C to 31 °C, the resonance dip red-shifted continually from 1537.57 nm to 1541.08 nm with the sensitivity of 3.51 nm/°C, which means that the resolution of the temperature probe can reach 0.005 °C using the experimental equipment in this paper (AQ6370, 600–1700 nm, resolution 20 pm, Yokogawa Electric Corp., Tokyo, Japan). Here, the temperature resolution of the proposed sensor was obtained by dividing the spectral resolution of the spectra analyzing equipment by the corresponding temperature sensitivity, that was (20 pm)/(3.51 nm/°C) ≈ 0.005 °C. For FBG sensors, the sensing parameters can be obtained by wavelength demodulation with the aid of commercial wavelength demodulator, whose wavelength resolution can reach up to 1 pm. It means that the temperature resolution of our proposed sensor is promising to be further improved to less than 0.0003 °C by using the commercial fiber demodulator.

**Figure 6:**
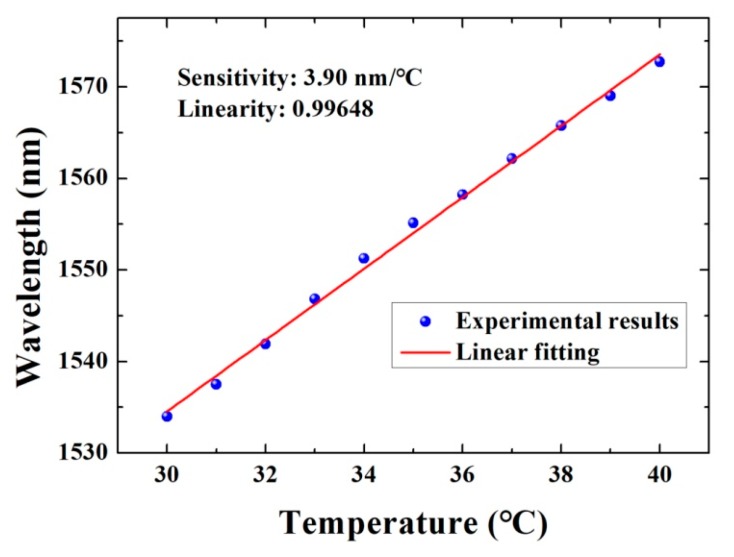
Temperature sensing characteristic curve of microfiber-microsphere temperature probe during 30–40 °C.

In Figure 6, it is shown a temperature sensing characteristic curve of the proposed fiber Fabry-Perot temperature probe. When the temperature was raised from 30 °C to 40 °C by a step of 1 °C, the average temperature sensitivity was determined as 3.90 nm/°C, and the linearity for the experimental data was 0.99648. This sensitivity is a little higher than the former sensitivity of 3.51 nm/°C during the temperature increasing process from 30 °C to 31 °C. This difference can be attributed to the uneven expansion of PDMS, resulting in a 10% uncertainty in the sensitivity. Here, a resonance dip was traced and its wavelength locations were recorded as the experimental data points. The working range will be limited by the FSR of the interference spectra, the output wavelength range of the light source, and the wavelength limitation of the spectra analyzer. For example, the light source has the output wavelength of 1520 nm to 1560 nm, which means that once a resonance dip is chosen, its wavelength shift can only be recorded no longer than 40 nm due to the high temperature sensitivity (3.90 nm/°C × 10 °C = 39 nm). However, the temperature sensing performance for a wider temperature change range is possible to be calibrated by recording the relative wavelength shift of the whole spectra using the different resonance dips in the particular wavelength ranges. For the actual application usage, the temperature sensing probe can work near a desired temperature point to monitor its fluctuation in an FSR.

## 4. Discussion

The temperature sensing performance of the Fabry-Perot (FP) interferometer fiber sensors reported in the references have been compared in Table 1. 

Comparing with the current state of the art of Fabry-Perot temperature sensors, the manufacturing process of the proposed temperature sensor is simple and convenient, the cost is low and the technology requirements are not very high. Its integrated reflector end enables its application in some special areas, especially for the high humidity environment. Since most open-end typed Fabry-Perot structures were usually constructed by coating film as the reflector. Compared with the common fiber Fabry-Perot temperature probe with open-cavities, the front section of the probe is closed, which can avoid the influence of ambient humidity on temperature sensing performance. In this paper, the influence of microfiber taper diameter and cavity length on the interference spectrum is studied, and the possibility of using it as a temperature sensing probe is preliminarily studied. Its long-term operational stability and repeatability need to be further verified by experiments.

Furthermore, it is desirable to obtain a spherical reflecting end face when a hollow core fiber is melted using a fusion splicer. However, it is found that, during the actual production process, it is difficult to obtain a perfect spherical structure by melting, but instead of a cone inner-face. When the central axis of the microfiber taper is not aligned with that of the hollow core fiber, the optical signal passing through the Fabry-Perot cavity is difficult to propagate along the axis, resulting in the multiple interference modes in the reflection spectrum. On the other hand, when the perfect spherical reflecting end face is prepared, the inner and outer surfaces of the spherical cavity will form another reflection, and the three beams of light propagating along the central axis of the fiber interfere to each other and generate the superposition of two interference spectra, forming a cursor effect. Since this effect can effectively improve the sensitivity of the sensor, it provides a new research direction for the sensing probe. 

## 5. Conclusions

In this paper, a microfiber taper was fabricated by flame scanning drawing method, and a spherical hollow fiber was obtained by arc discharge melt technique using a fiber fusion splicer. These two structures were later used to construct a microfiber Fabry-Perot structure. The impact of cavity length on the transmission spectra has been experimentally studied, where a shorter cavity length contributes to a smaller FSR and a sharper dip. Different formed PDMS solution and cured methods have been studied and compared to fill the air cavity. Finally, a microfiber Fabry-Perot structure with the cavity length of 41 μm and a microfiber diameter of 50 μm was fabricated. By filling with the PDMS with high thermo-optic and elastic-optic coefficients, a temperature probe was obtained and experimental demonstrated with a high temperature sensitivity of 3.9 nm/°C, compared to many highly sensitive Fabry-Perot temperature sensors proposed recently. The manufacturing process is simple and convenient, the cost is low and the technology requirements are not high. Comparing with the current state of the art of Fabry-Perot temperature sensors, it indicates the better sensing performance and it is expected to be applied in electromagnetic interference and humidity fields. Due to its high sensitivity and miniature structure, the proposed temperature probe will be used in precision medicine and fine chemistry as a promising candidate for monitoring the slight temperature fluctuation during biological interaction process and chemical reaction.

## Figures and Tables

**Table 1:** Sensing performance comparison for some typical Fabry-Perot temperature fiber sensors.

Structure or Materials	Sensitivity	Range	Reference
TiO_2_ film/PDMS overlay	0.13 dB/°C	22–60 °C	[4]
SMF/Capillary	−60.79 nW/°C	20–35 °C	[7]
Cascaded Fabry-Perot interferometers/Liquid crystal filling/Vernier effect	19.55 nm/°C	30–120 °C	[8]
Silicon/UV glue	84.4 pm/°C	−50–130 °C	[9]
Air cavity	9.91 pm/°C 15.88 pm/°C	100–400 °C 400–1100 °C	[10]
FBG/UV glue	81.0 pm/°C	−60–140 °C	[11]
Cr/Capillary/Ni jacket	~7.7 mrad/°C	−25–950 °C	[12]
Nafion film	2.71 nm/°C	−15–65 °C	[13]
Graphene films	352 pm/°C	20–60 °C	[14]
Photonics crystal fiber (PCF)	15.61 pm/°C	300–1200 °C	[15]
Microfiber tip	13.6 pm/°C	25–1000 °C	[16]
Gas filled capillaries	74.6 pm/°C	127–327 °C	[17]
Ho^3+^ doped fibers	75 pm/°C	20–50 °C	[18]
Liquid polymer	877 pm/°C	30–60 °C	[19]
Refractive index liquid/PCF	~14.72 nm/°C	18–21 °C	[20]
Microfiber/PDMS	11.86 nm/°C	43–50 °C	[21]
Microfiber/Microsphere/PDMS	3.9 nm/°C	30–40 °C	This work
